# 374. A Multicenter Open-Label Single-Arm Clinical Trial of Combination Therapy of Surgery, Itraconazole, Doxycycline, and Azithromycin for Vascular Pythiosis

**DOI:** 10.1093/ofid/ofad500.444

**Published:** 2023-11-27

**Authors:** Kasama Manothummetha, Pattama Torvorapanit, Nuttapon Susaengrat, Navaporn Worasilchai, Ariya Chindamporn, Nipat Chuleerarux, Ratiporn Bansong, Watchara Wattanasoontornsakul, Petchdee Oranrigsupak, Jaruwan Diewsurin, Paruspak Paruspak Payoong, Prasopchai Kongsakpaisan, Ginthasuphang Wangsapthawi, Nirada Siriyakorn, Achitpol Thongkam, Sureerat Watcharasuwanseree, Jaedvara Thanakitcharu, Surachai Leksuwankun, Tanaporn Meejun, Kasidis Phongkhun, Nattapong Langsiri, Parichart Sakulkonkij, Sunee Chayangsu, Kanokwan Laohasakprasit, Bhoowit Lerttiendamrong, Supphachoke Khemla, Karan Srisurapanont, Rattagan Kajeekul, Poom Chayapum, Rongpong Plongla, Nitipong Permpalung

**Affiliations:** Infectious Disease, The Johns Hopkins University School of Medicine, Glen Burnie, Maryland; Chulalongkorn University and King Chulalongkorn Memorial Hospital, Bangkok, Krung Thep, Thailand; Khonkaen Hospital, Khon Kaen, Khon Kaen, Thailand; Chulalongkorn university, ฺBangkok, Krung Thep, Thailand; Faculty of Medicine, Chulalongkorn University, Bangkok, Krung Thep, Thailand; Jackson Memorial Hospital/University of Miami, Miami, Florida; Sappasithiprasong hospital, Ubonratchathani, Buriram, Ubon Ratchathani, Thailand; Maharat Nakhon Ratchasima Hospital, Nakhon Ratchasima, Nakhon Ratchasima, Thailand; Nan hospital, Nan, Nan, Thailand; Buddhachinaraj hospital, Muang, Phitsanulok, Thailand; Chiangrai Prachanukroh Hospital, Chiang Rai, Chiang Rai, Thailand; Nakhon Pathom hospital, Nakhon Pathom, Nakhon Pathom, Thailand; Chiangrai Prachanukroh Hospital, Chiang Rai, Chiang Rai, Thailand; Department of Medicine, Rajavithi Hospital, Bangkok, Krung Thep, Thailand; Chulalongkorn university, ฺBangkok, Krung Thep, Thailand; Udon Thani Hospital, Meung, Udon Thani, Thailand; The Faculty of Medicine Srinakharinwirot University, Bangkok, Krung Thep, Thailand; Chulalongkorn university, ฺBangkok, Krung Thep, Thailand; Faculty of Medicine Chiang Mai University, Chiang Mai, Chiang Mai, Thailand; Chulalongkorn university, ฺBangkok, Krung Thep, Thailand; Chulalongkorn university, ฺBangkok, Krung Thep, Thailand; Chiang Mai University, Chiang Mai, Chiang Mai, Thailand; Surin hospital, Surin, Surin, Thailand; Sawanpracharak hospital, Nakhonsawan, Nakhon Sawan, Thailand; Chulalongkorn university, ฺBangkok, Krung Thep, Thailand; Nakhon-Phanom hospital, Nakorn Phanom, Nakhon Phanom, Thailand; Faculty of Medicine Chiang Mai university, Chiang Mai, Chiang Mai, Thailand; Maharat Nakhon Ratchasima Hospital, Nakhon Ratchasima, Nakhon Ratchasima, Thailand; Paholpolpayuhasena Hospital, Kanchanaburi, Kanchanaburi, Thailand; Chulalongkorn university, ฺBangkok, Krung Thep, Thailand; Johns Hopkins University School of Medicine, Baltimore, MD

## Abstract

**Background:**

Vascular pythiosis, caused by *Pythium insidiosum*, is a neglected, life-threatening condition which carried a 100% mortality rate, within 3 months, among patients with residual disease after surgery, regardless of antifungal treatment. Recently, Thai *P. insidiosum* isolates were found to susceptible to doxycycline and azithromycin, with evidence of synergy. We report results of a clinical trial from 15 centers in Thailand (Thai Clinical Trial Registry number TCTR20191217006).

**Methods:**

This study enrolled patients with vascular pythiosis who had 2 of the 4 diagnostic criteria: consistent clinical presentation, positive serum *P. insidiosum-*specific antibodies, positive cultures, or positive histopathology. All received surgery, itraconazole, doxycycline, and azithromycin. Patients were followed up until 6 months after diagnosis. Subsequent follow-ups after 6 months were at the clinician’s discretion if there were any concerns for residual diseases or unresectable arterial lesions.

**Results:**

40 patients were enrolled, with a median age (interquartile range [IQR]) of 51 (45-56) years. 38 (95%) had major thalassemia, 1 (2.5%) had liver cirrhosis, and 1 (2.5%) had myeloproliferative syndrome. None had known immunocompromised conditions. After surgery, 14 (35%) still had residual diseases. At 6 months after diagnosis, 3 (7.5%) patients died (disseminated pythiosis, sepsis with multi-organ failure, and ESBL *Klebsiella pneumoniae* bacteremia). Among patients with residual disease, 1 (7.1%) died and 13 are still alive. Median follow-up time (IQR) among patients with residual disease was 385 (209-622) days. There were no statistical differences in time from onset to surgery and time from onset to first antimicrobial between patients with and without residual disease after surgery (Table). One patient had to stop doxycycline prematurely due to photosensitivity.

Patient characteristics

**Figure:**
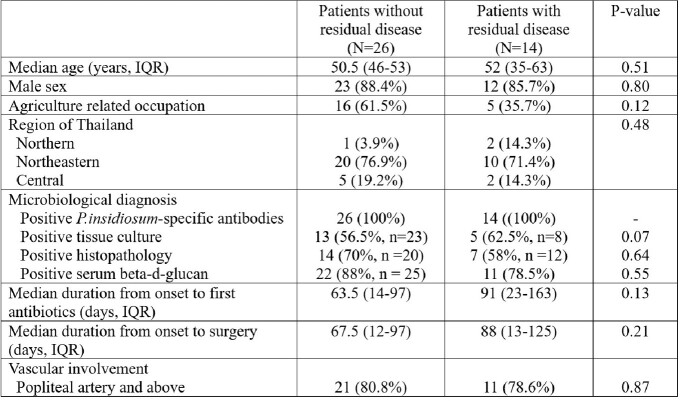

IQR: interquartile range; N: number

**Conclusion:**

The new treatment combination with surgery, itraconazole, azithromycin, and doxycycline improved survival among patients with vascular pythiosis who had residual disease.

**Disclosures:**

**Nitipong Permpalung, MD, MPH**, Alcimed: Advisor/Consultant|CareDx: Grant/Research Support|Cidara Therapeutics: Grant/Research Support|Clarion: Advisor/Consultant|ClearView: Advisor/Consultant|IMMY Diagnostics: Grant/Research Support|Merck: Grant/Research Support|Scynexis: Grant/Research Support

